# Supplementary material for: Influence of Temperature on Exciton Dynamic Processes in CuPc/C60 Based Solar Cells
Source: Micromachines (Basel). 2021 Oct 22;12(11):1295. doi: 10.3390/mi12111295 (PMC8625268; doi:10.3390/mi12111295)
Supplement: Supplementary file 1 [file micromachines-12-01295-s001.zip › micromachines-1410719-supplementary.pdf]

Influence of Temperature on Exciton Dynamic Processes in a CuPc/C60 Based Solar Cell

Lijia Chen [1,†], Lun Cai [2,†], Lianbin Niu [1], Pan Guo [1] and Qunliang Song [3,*]

[1] College of Physics and Electronics Engineering, Chongqing Normal University, Chongqing 401331, China; ljchen01@cqnu.edu.cn (L.C.); niulb03@126.com (L.N.); guopan@cqnu.edu.cn (P.G.)

[2] School of Materials, Sun Yat-sen University, Shenzhen 518107, China; cailun@mail2.sysu.edu.cn

[3] Institute for Clean Energy and Advanced Materials, School of Materials and Energy, Southwest University, Chongqing 400715, China

* Correspondence: qlsong@swu.edu.cn

† These authors contributed equally to this work.

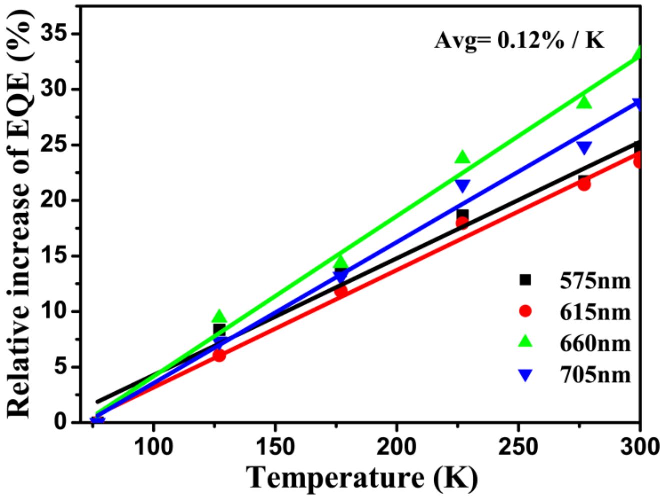

Figure S1. The relative increase of EQE with temperature at different wavelengths

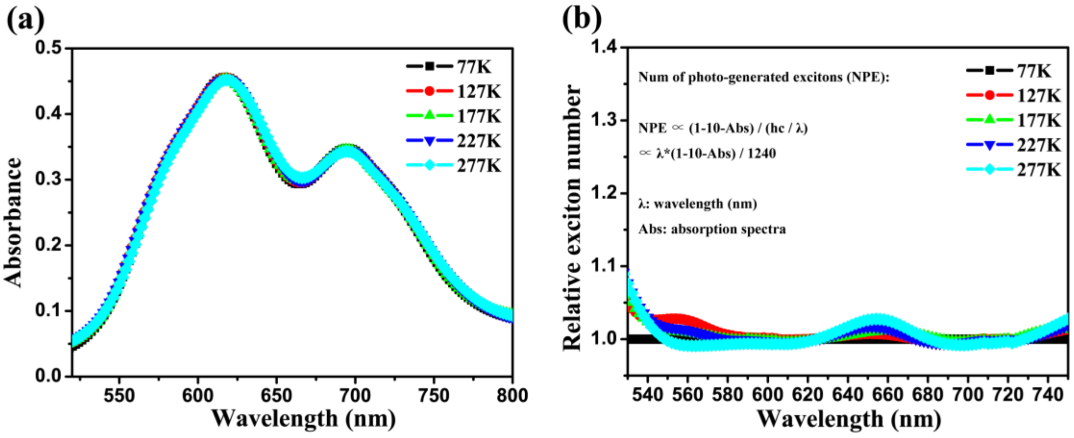

Figure S2. (**a**)Absorption spectra for a 50 nm CuPc thin film thermal evaporated on quartz substrate and (**b**) the relative changes of absorption efficiency.

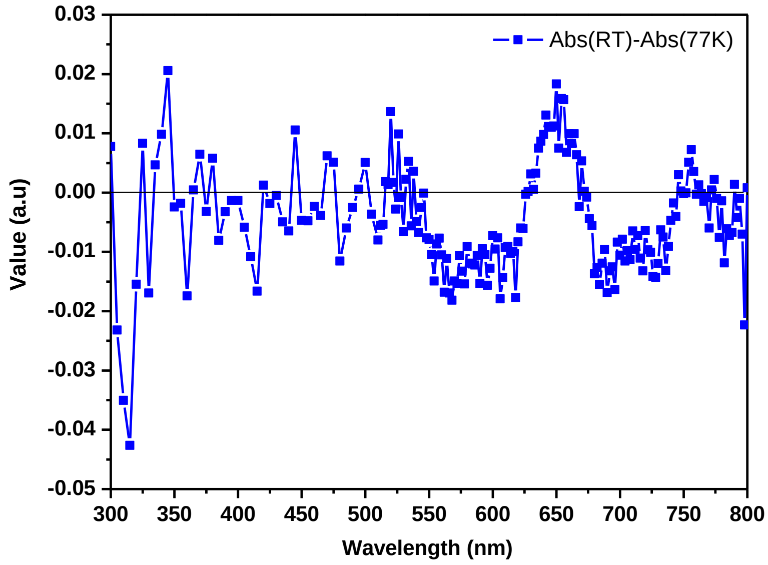

Figure S3. Absorption ratio with 77 K and 300 K.
